# Quantitative trait locus mapping of fruit aroma compounds in cucumber (*Cucumber sativus* L.) based on a recombinant inbred line population

**DOI:** 10.1093/hr/uhac151

**Published:** 2022-07-06

**Authors:** Yinhui Sun, Xvzhen Li, Zhaoyang Ma, Shuxia Chen

**Affiliations:** College of Horticulture, Northwest A&F University, Yangling 712100, China; College of Horticulture, Northwest A&F University, Yangling 712100, China; College of Horticulture, Northwest A&F University, Yangling 712100, China; College of Horticulture, Northwest A&F University, Yangling 712100, China; Shaanxi Engineering Research Center for Vegetables, Yangling 712100, China

## Abstract

The fresh and unique flavor of cucumber fruits, mainly composed of aldehydes and alcohols, is one of its most important fruit qualities. However, little is known about the genetic basis of aroma compounds in cucumber fruit and the related quantitative trait loci (QTLs). In this study, genomic screening of QTLs underlying aroma compounds was performed based on the genetic linkage map constructed using 1301 single-nucleotide polymorphism (SNP) markers from genotyping-by-sequencing of a recombinant inbred line (RIL) population developed from Q16 × Q24. Significant genetic variations of aroma compounds in the RIL population were observed, and a total of 28 QTLs were screened. A major QTL (*qol8-2.1*) related to (*E*,*Z*)-2,6-nonadien-1-ol was detected with a markedly high LOD score (10.97 in 2020 and 3.56 in 2019) between mk190 and mk204 on chromosome 2. Genome scans identified a cluster of nine lipoxygenase genes in this region. A significant positive correlation was detected between *CsaV3_2G005360* (*CsLOX08*) and (*E*,*Z*)-2,6-nonadien-1-ol, and five amino acid variations were detected between the CsLOX08 protein sequences of the two parental lines. Based on the genome variation of CsLOX08, we developed an InDel marker. Genotyping of InDel markers was consistent with the content of (*E*,*Z*)-2,6-nonadien-1-ol in RILs, which were also verified in nine cucumber inbred lines. The results will give breeders guidance for obtaining better flavor in cucumber.

## Introduction

Cucumber is cultivated and consumed worldwide, and is valued by consumers for its fresh and unique flavor, especially for eating raw [1]. More than 70 aroma compounds have been identified in cucumber fruit, including aldehydes, alcohols, ketones, terpenes, esters, and furans, which all contribute to the typical flavor of cucumber fruits [2, 3]. Of these, C6 and C9 aldehydes and alcohols are the main compounds of cucumber flavor [4, 5]. With the fast development of aroma measurement methods, solid-phase microextraction (SPME) combined with gas chromatography–mass spectrometry (GC–MS) has been proved to be an effective method for the qualitative and quantitative analysis of aroma compounds [6]. More aroma compounds have been detected, and measurements of aroma compounds have become more convenient and accurate.

Unsaturated fatty acid-derived C6 and C9 aldehydes and alcohols were synthesized and metabolized through the lipoxygenase pathway [7]. Lipoxygenase was the first key enzyme in the lipoxygenase pathway, which introduces molecular oxygen at C13 or C9 to form 9/13-hydroperoxy-octadecadienoic acid (9/13 HPOD) or 9/13-hydroperoxy-octadecatrienoic acid (9/13 HPOT). Then 9/13 HPOD and HPOT are cleaved by hydroperoxide lyase (HPL) to form C6 or C9 aldehydes [8]. Therefore, there are two types of lipoxygenase (9-LOX and 13-LOX) in plants. The C6 and C9 aldehydes are reduced to the corresponding C6 and C9 alcohols by alcohol dehydrogenase (ADH) [9, 10].

C6 aroma compounds mainly consist of compounds such as (*E*)-2-hexenal, hexanol, (*E*)-2-hexene-1-ol, and (*E*)-3-hexene-1-ol, and C9 aroma compounds mainly consist of (*E*,*Z*)-2,6-nonadienal, (*E*)-6-nonenal, (*Z*)-6-nonenal, nonanal, (*Z*)-6-nonen-1-ol, (*E*,*Z* )-2,6-nonadien-1-ol, and (*E*,*Z* )-3,6-nonadien-1-ol. The kinds and contents of aroma compounds vary significantly among varieties [11–13]. C6 aroma compounds give cucumber fruits distinctive grassy, green aromas, and C9 aroma compounds provide a cucumber-like note [12, 14]. Cucumber fruits contain higher amounts of C9 aroma compounds, and the content of C6 aroma compounds in leaves and young fruits is higher than in other parts of the cucumber [3].

It was reported that the content of aroma compounds in cucumber fruit was influenced by multiple genes and the environment. The heritability of characteristic aroma compounds in cucumber fruit was low, indicating that the content of aroma compounds was affected dramatically by environmental factors [15]. Understanding the genetics of aroma compounds is highly relevant for flavor breeding [16]. The genetics of fruit aroma compounds in plants has been demonstrated in several studies. Quantitative trait loci (QTL) related to the content of *S*-methyl-thioacetate in melon fruits were investigated by QTL mapping in a recombinant inbred line (RIL) population, and the *CmThAT1* gene was identified as a candidate gene [17]. A total of 166 QTLs for 82 different volatile organic compounds were identified based on a melon RIL population [18]. In tomato, a total of 102 QTLs for 39 aroma compounds were located on different chromosomes using 169 RIL populations [19]. A major QTL locus associated with the content of phenylalanine-derived volatiles in tomato fruit was mapped on chromosome 4. Fine mapping showed that *FLORAL4* was a candidate gene, and the gene function was verified using the CRISPR-Cas9 system [20]. QTL mapping of fruit aroma compounds has also been studied in some fruit trees. In apple, *MdoOMT1* and *AAT1* were identified as candidate genes for methylated phenylpropenes and esters, respectively, by QTL mapping [21, 22]. A cluster of terpene synthase (*TPS*) genes was identified at a QTL associated with the content of 1,8-cineole on linkage group 29a in kiwifruit, and *AcTPS1b* was confirmed as a critical gene for the production of 1,8-cineole by enzyme character analysis and gene expression analysis [23]. However, few reports have identified the loci that control cucumber aroma compounds using QTL analysis.

In this study, QTL mapping of cucumber fruit aroma compounds was performed using 129 RILs developed by crossing lines Q16 and Q24. A major QTL (*qol8-2.1*) related to (*E*,*Z*)-2,6-nonadien-1-ol was screened, and nine lipoxygenase genes were identified in this QTL region. Correlation analysis between (*E*,*Z*)-2,6-nonadien-1-ol content and expressions of nine *CsLOX*s was carried out, and sequence analysis verified that *CsaV3_2G005360* (*CsLOX08*) might be the candidate gene*.* An InDel marker was developed based on the genome variation of *CsLOX08*, and the InDel marker could genotype the high and low (*E*,*Z*)-2,6-nonadien-1-ol content individuals in cucumber fruits, which will provide cucumber breeders with some basis for molecular breeding.

## Results

### Phenotypic variation of aroma compounds

The aroma compound phenotypes of the two parental lines were determined in two years. The result revealed that the total content of C6 aroma compounds (six-carbon aldehydes and alcohols) and C9 aroma compounds (nine-carbon aldehydes and alcohols) was significantly higher in line Q16 than in line Q24 (Table 1). The content of most aroma compounds of the two parental lines overlapped in both years. The difference in aroma compounds between two parental lines was analyzed in the two years. The contents of 2-hexanal, (*E*)-4-nonenal, and (*E*,*Z*)-2,6-nonadien-1-ol were stably and significantly higher in Q16 than in Q24 in both years. However, the differences in some aroma compounds between the two parental lines were inconsistent between the two years, such as (*Z*)-3-hexen-1-ol, (*Z*,*Z*)-3,6-nonadienal, (*E*)-2-nonenal, (*Z*)-3-nonen-1-ol, and (*E*)-6-nonen-1-ol.

**Table 1:** Content of the C6 and C9 aroma compounds (μg/g fresh weight) in fruits of parental lines and RILs in the two study years

Aroma compound	Parental line				RIL population			
	2019		2020		2019		2020	
Q16	Q24	Q16	Q24	Mean ± SD	Range	Mean ± SD	Range	
*C6 aldehydes*								
Hexanal (al1)	0.272 ± 0.072	0.196 ± 0.020	**0.239 ± 0.012**	**0.285 ± 0.008**	**0.369 ± 0.132**	**0.164–0.829**	0.243 ± 0.093	0.146–0.848
2-Hexenal (al2)	**0.314 ± 0.039**	**0.166 ± 0.024**	**0.490 ± 0.042**	**0.254 ± 0.046**	**0.329 ± 0.111**	**0.155–0.641**	0.297 ± 0.088	0.143–0.614
*C6 alcohols*								
1-Hexanol (ol1)	0.048 ± 0.017	0.019 ± 0.010	0.312 ± 0.083	0.449 ± 0.164	0.094 ± 0.088	0.009–0.472	0.420 ± 0.301	0.102–1.532
(*Z*)-3-Hexen-1-ol (ol2)	**0.006 ± 0.001**	**0.002 ± 0.001**	**0.007 ± 0.001**	**0.010 ± 0.001**	0.034 ± 0.030	0.002–0.132	0.014 ± 0.009	0.002–0.047
*C9 aldehydes*								
Nonanal (al3)	0.100 ± 0.014	0.089 ± 0.005	0.267 ± 0.050	0.241 ± 0.035	**0.126 ± 0.053**	**0.038–0.270**	0.182 ± 0.072	0.080–0.381
(*E*)-4-Nonenal (al4)	**0.005 ± 0.001**	**0.003 ± 0.001**	**0.030 ± 0.007**	**0.015 ± 0.004**	0.043 ± 0.110	0.002–0.603	0.016 ± 0.009	0.002–0.050
(*E*)-6-Nonenal (al5)	0.348 ± 0.058	0.319 ± 0.025	0.876 ± 0.140	0.749 ± 0.072	**0.514 ± 0.222**	**0.158–1.187**	0.601 ± 0.259	0.190–1.224
(*Z*)-2-Nonenal (al6)	0.007 ± 0.001	0.008 ± 0.001	nd	nd	0.015 ± 0.007	0.004–0.033	nd	nd
(*Z*,*Z*)-3,6-Nonadienal (al7)	0.025 ± 0.004	0.020 ± 0.001	**0.040 ± 0.002**	**0.013 ± 0.001**	**0.051 ± 0.023**	**0.010–0.122**	**0.027 ± 0.011**	**0.007–0.052**
(*E*)-2-Nonenal (al8)	0.792 ± 0.099	0.844 ± 0.076	**2.371 ± 0.169**	**1.494 ± 0.142**	**1.441 ± 0.648**	**0.415–3.230**	1.723 ± 0.678	0.639–4.362
(*E*,*Z*)-2,6-Nonadienal (al9)	2.845 ± 0.336	2.317 ± 0.126	4.022 ± 0.606	3.142 ± 0.225	5.089 ± 2.107	1.261–12.348	3.579 ± 1.223	1.509–8.431
(*E*,*E*)-2,4-Nonadienal (al10)	0.005 ± 0.0001	0.004 ± 0.0002	0.038 ± 0.006	0.040 ± 0.001	0.008 ± 0.003	0.003–0.025	0.038 ± 0.012	0.019–0.083
*C9 alcohols*								
1-Nonanol (ol3)	0.047 ± 0.009	0.042 ± 0.0002	**0.167 ± 0.014**	**0.214 ± 0.022**	0.140 ± 0.108	0.003–0.604	0.146 ± 0.095	0.016–0.469
(*Z*)-3-Nonen-1-ol (ol4)	**0.053 ± 0.005**	**0.040 ± 0.005**	0.187 ± 0.025	0.200 ± 0.019	0.130 ± 0.078	0.017–0.363	0.157 ± 0.069	0.053–0.391
2-Nonen-1-ol (ol5)	**0.058 ± 0.010**	**0.031 ± 0.004**	**0.061 ± 0.005**	**0.014 ± 0.002**	0.133 ± 0.137	0.006–0.819	0.097 ± 0.081	0.008–0.445
(*E*)-6-Nonen-1-ol (ol6)	0.182 ± 0.039	0.134 ± 0.013	**0.568 ± 0.009**	**0.649 ± 0.028**	0.553 ± 0.436	0.038–3.161	0.524 ± 0.341	0.048–1.649
(*E*,*Z*)-3,6-Nonadien-1-ol (ol7)	**0.104 ± 0.010**	**0.063 ± 0.011**	**0.341 ± 0.037**	**0.240 ± 0.010**	0.278 ± 0.173	0.045–0.932	0.242 ± 0.109	0.073–0.580
(*E*,*Z*)-2,6-Nonadien-1-ol (ol8)	**0.206 ± 0.056**	**0.045 ± 0.012**	**0.168 ± 0.006**	**0.020 ± 0.007**	0.476 ± 0.582	0.047–4.260	0.252 ± 0.231	0.006–1.102
(*Z*)-6-Nonen-1-ol (ol9)	**0.006 ± 0.001**	**0.002 ± 0.0009**	nd	nd	0.016 ± 0.013	0.002–0.060	nd	nd
Total	5.422 ± 0.641	4.344 ± 0.307	**10.184 ± 0.440**	**8.029 ± 0.663**	9.688 ± 3.583	3.600–20.355	8.401 ± 2.422	4.340–16.326

Aroma compounds significantly different between Q16 and Q24 are in bold type in the parental lines columns (*P* < .05; Student’s test), and aroma compounds with normal distributions in the RIL population are in bold type in the RIL population columns (*P* < .05; Shapiro–Wilk test).

Nineteen C6 and C9 aroma compounds were chosen to be analyzed for phenotypic variation in the RIL population in the two years, including hexanal, 2-hexenal, 1-hexanol, (*Z*)-3-hexen-1-ol, (*E*,*Z*)-2,6-nonadienal, (*E*)-2-nonenal, (*E*)-6-nonenal, nonanal, (*Z*, *Z*)-3,6-nonadienal, (*E*,*E*)-2, 4-nonadienal, (*E*)-4-nonenal, (*Z*)-2-nonenal, (*E*)-6-nonen-1-ol, (*E*,*Z*)-2,6-nonadien-1-ol, (*Z*)-3-nonen-1-ol, (*E*,*Z*)-3,6-nonadien-1-ol, 2-nonen-1-ol, 1-nonanol, and (*Z*)-6-nonen-1-ol (Table 1). The total contents of C6 and C9 aroma compounds varied greatly among RILs, ranging from 3.600 to 20.355 μg/g fresh weight (FW). Significant variations of aroma compounds were found in the RIL population. The largest variation was that of (*E*,*Z*)-2,6-nonadienal, which was the most abundant compound in the cucumber fruits, ranging from 1.261 to 12.348 μg/g FW in the RIL population in both years, followed by (*E*)-2-nonenal, which ranged from 0.415 to 4.362 μg/g FW. The smallest variations were of (*E*,*E*)-2, 4-nonadienal and (*Z*)-3-hexen-1-ol, ranging from 0.003 to 0.083 μg/g FW and from 0.002 to 0.132 μg/g FW, respectively, in the RIL population, indicating that different aroma compounds had differed in heritability and the extent to which they were affected by environmental factors.

**Figure 1:**
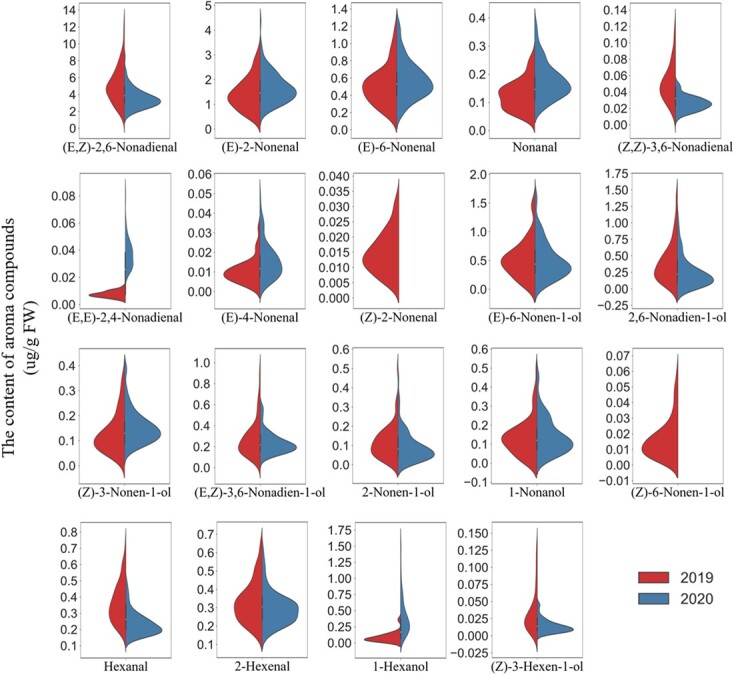
Violin plot distribution of C6 and C9 aroma compounds in the RIL population in 2019 (left side of plot) and 2020 (right side).

The frequency distribution of aroma compounds in the RIL population in the two years is illustrated in Fig. 1. Most aroma compounds exhibited a normal or a near-normal distribution in the RIL population (Fig. 1). This suggests that these aroma compounds are quantitative traits. The violin plots showed that most aroma compounds were stable in the two years except for a few of them, such as (*E*,*E*)-2, 4-nonadienal, (*Z*)-2-nonenal, (*Z*)-6-nonen-1-ol, and 1-hexanol.

### Multivariate analysis and correlations among different aroma compounds

**Figure 2:**
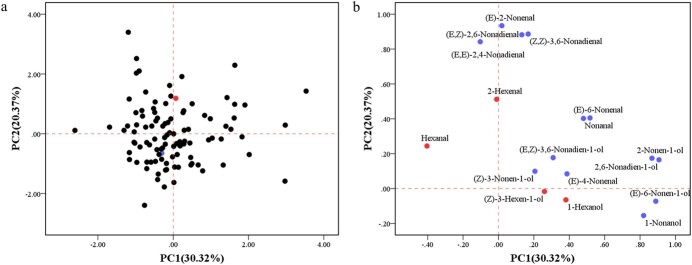
PCA of C6 and C9 aroma compounds determined in the RIL population in 2020. **a** Points show the PCA scores of each line, Q16 and Q24, highlighted in red and blue, respectively. **b** Loading plots of C6 and C9 aroma compounds.

To determine the main variables accounting for the phenotypic variation of aroma compounds in the RIL population, principal component analysis (PCA) was employed to perform multivariate analysis. The first two principal components, PC1 and PC2, accounted for 30.32 and 20.37% of the total phenotypic variation (Fig. 2). The RIL population was distributed evenly without forming clear clusters. The two parental lines were clearly separated from each other, indicating that they contain relatively different aroma compound profiles. The variables that positively contributed to the first principal component were 2-nonen-1-ol, (*E*,*Z*)-2,6-nonadien-1-ol, (*E*)-6-nonen-1-ol, and 1-nonanol, and the variables (*E*)-2-nonenal, (*E*,*Z*)-2,6-nonadienal, (*Z*,*Z*)-3,6-nonadienal, and (*E*,*E*)-2, 4-nonadienal showed the highest and the most contrasting eigenvalues in the second principal component.

**Figure 3:**
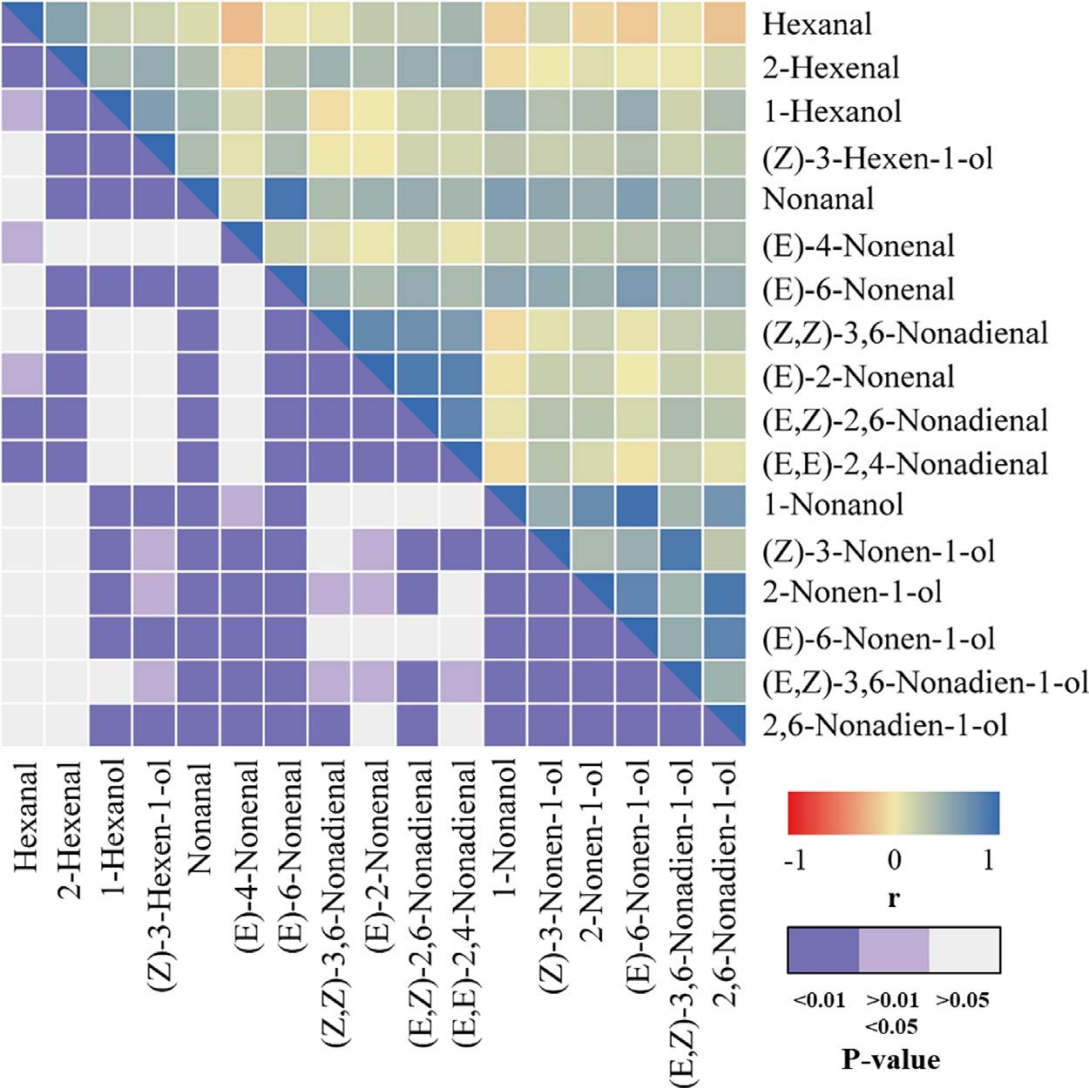
Correlation heat map showing correlations among C6 and C9 aroma compounds measured across RILs population. The correlation coefficient (*r*) and Bonferroni-corrected *P* values are shown in the lower right corner.

The correlations between different aroma compounds were calculated and are depicted in Fig. 3 and Supplementary Data Tables S1 and S2. It is noteworthy that significant positive correlations (*P* < .01) were observed between (*E*,*Z*)-2,6-nonadienal and (*E*,*Z*)-2,6-nonadien-1-ol in 2019 (*r* = 0.604) and 2020 (*r* = 0.293). In addition, there was a significant positive correlation between (*E*)-6-nonenal and (*E*)-6-nonen-1-ol in 2019 (*r* = 0.654) and 2020 (*r* = 0.634), indicating that there was a significant correlation between aldehydes and their corresponding alcohols. The strongest positive correlations were found between (*E*)-6-nonen-1-ol and (*E*,*Z*)-2,6-nonadien-1-ol (*r* = 0.95) and between nonanal and (*E*)-6-nonenal (*r* = 0.91).

### Genotyping by sequencing of RIL population and construction of a high-density linkage map

**Figure 4:**
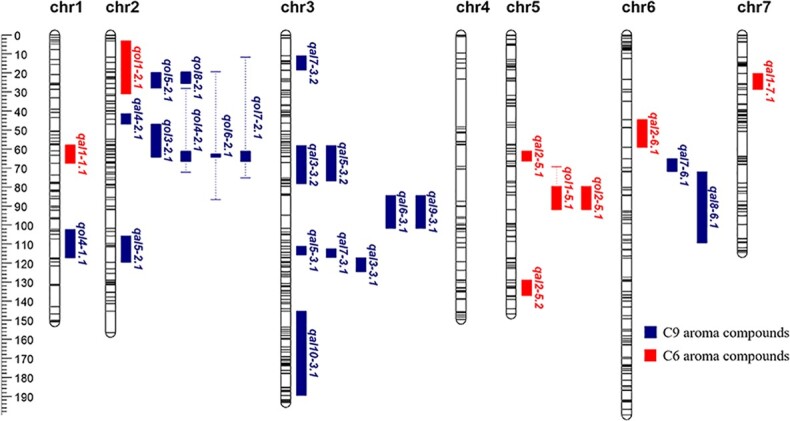
Genetic positions of QTLs controlling C6 and C9 aroma compounds detected in 2019 and 2020. 1.5-LOD QTL intervals are drawn at the right of each linkage group.

The RIL population and two parental lines were re-sequenced using the genotyping-by-sequencing approach. A total of 70.23 G clean bases with high-quality (Q20 ≥ 96.16% and Q30 ≥ 89.32%) and 484.90 Mb clean reads were obtained by aligning with the cucumber reference genome 9930 V3. Based on the genotyping results of two parental lines, 241 166 single-nucleotide polymorphisms (SNPs) were found, and a total of 163 900 SNPs were identified among the RIL population. After deleting segregation distortion (*P* < .001), we obtained 1301 effective SNPs. Based on 1301 SNPs, a high-density SNP linkage map of the RIL population was constructed, which contained 1301 SNP markers distributed in seven linkage groups and covered a total genetic distance of 1111.41 cM with 0.85 cM per marker (Fig. 4, Supplementary Data Table S3).

### QTL mapping of C6 and C9 aroma compounds in the RIL population

We performed QTL analysis of C6 and C9 aroma compounds with a 2-year phenotypic dataset from the RIL population based on the linkage maps constructed in this study. The CIM calculations were performed for whole-genome scanning of QTLs based on the phenotype data of aroma compounds. A total of 28 QTLs were screened for 19 aroma compounds and distributed on six chromosomes, except for chromosome 4 (Fig. 4, Table 2). The QTLs explained 2.11–39.30% of the phenotype variance of aroma compounds (Table 2). Most QTLs were distributed on chromosomes 2, 3, and 5. Among them, four QTLs associated with C6 aldehydes and alcohols were located on chromosome 5, nine QTLs associated with C9 aldehydes were located on chromosome 3, and six QTLs related to C9 alcohols were located on chromosome 2.

**Table 2:** QTLs identified in the RIL population.

Trait	QTL name	Environment	Chromosome	LOD value	*R* ^2^ (%)	Additive	Peak position	Nearest marker	Marker interval	Confidence interval
Hexanal (al1)	*qal1-1.1*	2019	1	4.29	16.23	4.06	63.42	mk99	mk90-mk101	57.93–67.52
	*qal1-7.1*	2019	7	3.15	11.85	−3.50	27.80	mk1563	mk1546-mk1561	20.43–28.59
2-Hexenal (al2)	*qal2-5.1*	2019	5	9.19	39.30	7.30	62.60	mk1121	mk1119-mk1154	61.07–66.48
	*qal2-5.2*	2020	5	3.37	10.10	3.32	132.57	mk1194	mk1215-mk1172	128.84–137.00
	*qal2-6.1*	2019	6	3.07	10.14	−3.66	50.00	mk1327	mk1325-mk1341	44.56–59.08
1-Hexanol (ol1)	*qol1-2.1*	2020	2	3.48	2.11	−1.46	25.64	mk202	mk209-mk257	3.09–31.13
	*qol1-5.1*	2020	5	3.17	4.88	2.26	84.20	mk1184	mk1124-mk1207	69.33–91.87
		2019	5	5.78	23.36	4.98	84.20	mk1184	mk1168-mk1207	79.61–91.87
(*Z*)-3-Hexen-1-ol (ol2)	*qol2-5.1*	2019	5	5.06	24.40	5.16	85.00	mk1185	mk1168-mk1207	79.61–91.87
		2020	5	4.03	14.54	4.22	85.00	mk1185	mk1168-mk1207	79.61–91.87
Nonanal (al3)	*qal3-3.1*	2019	3	3.03	10.07	−2.93	121.02	mk741	mk714-mk747	117.32–124.59
	*qal3-3.2*	2020	3	3.36	5.58	2.33	60.00	mk590	mk585-mk641	58.15–78.33
(*E*)-4-Nonenal (al4)	*qal4-2.1*	2019	2	4.98	8.03	−2.61	44.38	mk285	mk283 -mk286	41.44–46.87
(*E*)-6-Nonenal (al5)	*qal5-2.1*	2020	2	3.07	7.84	2.89	110.54	mk407	mk401-mk410	105.66–119.53
	*qal5-3.1*	2019	3	5.22	12.58	−3.35	112.64	mk711	mk713-mk721	111.21–115.71
	*qal5-3.2*	2020	3	3.48	6.30	2.50	60.69	mk588	mk585-mk602	58.15–76.75
(*Z*)-2-Nonenal (al6)	*qal6-3.1*	2019	3	3.76	10.86	−3.17	95.00	mk688	mk664-mk676	84.54–101.79
(*Z*,*Z*)-3,6-Nonadienal (al7)	*qal7-3.1*	2019	3	3.25	10.98	−3.08	115.00	mk721	mk711-mk715	112.64–116.98
	*qal7-3.2*	2020	3	3.71	13.60	3.97	15.17	mk473	mk468-mk501	11.03–18.58
	*qal7-6.1*	2020	6	3.25	5.86	−2.60	69.92	mk1354	mk1344-mk1394	65.18–71.80
(*E*)-2-Nonenal (al8)	*qal8-6.1*	2019	6	3.19	8.35	−2.66	80.23	mk1360	mk1390-mk1409	72.00–109.37
(*E*,*Z*)-2,6-Nonadienal (al9)	*qal9-3.1*	2019	3	3.93	16.44	−4.21	95.00	mk688	mk664 -mk676	84.54–101.79
(*E*,*E*)-2,4-Nonadienal (al10)	*qal10-3.1*	2019	3	3.85	7.41	−2.49	155.89	mk794	mk788-mk879	145.2–189.62
1-Nonanol (ol3)	*qol3-2.1*	2020	2	3.67	8.25	2.97	62.96	mk293	mk286-mk304	46.87–64.35
(*Z*)-3-Nonen-1-ol (ol4)	*qol4-1.1*	2019	1	3.13	12.66	3.64	103.06	mk141	mk140-mk163	102.29–117.34
	*qol4-2.1*	2019	2	3.95	11.82	3.94	70.00	mk348	mk223-mk350	28.16–72.18
		2020	2	3.12	12.18	3.79	62.96	mk303	mk292-mk328	61.09–66.58
2-Nonen-1-ol (ol5)	*qol5-2.1*	2020	2	3.87	12.93	−3.82	23.28	mk198	mk190-mk227	19.80–28.04
(*E*)-6-Nonen-1-ol (ol6)	*qol6-2.1*	2019	2	3.05	5.90	2.21	62.96	mk303	mk191 -mk376	19.37–86.57
		2020	2	4.62	9.12	3.14	62.96	mk303	mk298-mk304	62.46–64.35
(*E*,*Z*)-3,6-Nonadien-1-ol (ol7)	*qol7-2.1*	2019	2	3.04	7.02	2.65	69.33	mk334	mk216-mk359	11.84–75.14
		2020	2	4.63	14.19	4.11	62.96	mk303	mk292-mk328	61.09–66.58
(*E*,*Z*)-2,6-Nonadien-1-ol (ol8)	*qol8-2.1*	2019	2	3.56	11.76	−3.22	20.00	mk194	mk191-mk204	19.37–25.53
		2020	2	10.97	17.53	−4.57	23.33	mk199	mk190-mk204	19.80–25.53

For C6 aroma compounds, eight QTLs, comprising *qal1-1.1*, *qal1-7.1*, *qal2-5.1*, *qal2-5.2*, *qal2-6.1*, *qol1-2.1*, *qol1-5.1*, and *qol2-5.1*, were identified for hexanal, 2-hexenal, 1-hexanol, and (*Z*)-3-hexen-1-ol (Fig. 4, Table 2), among which *qol1-5.1* and *qol2-5.1*, associated with 1-hexanol and (*Z*)-3-hexen-1-ol, were detected in the two seasons, mainly located between mk1124 and mk1207 on chromosome 5; four QTLs (*qal1-1.1*, *qal1-7.1*, *qal2-5.1*, and *qal2-6.1*) associated with hexanal and 2-hexenal were detected in 2019; and two QTLs (*qal2-5.2* and *qol1-2.1*) associated with 2-hexenal and 1-hexanol were detected in 2020. The LOD (logarithm of the odds) score of QTL *qal2-5.1* associated with 2-hexenal was the highest; it was detected in 2019, and explained 39.30% of the phenotypic variation for 2-hexenal, and was located between mk1119 and mk1154 on chromosome 5. The LOD score of QTL *qol1-5.1* associated with 1-hexanol was the next; it explained 23.36 and 4.88% of the phenotypic variation for 1-hexanol in 2019 and 2020, respectively, and was located between mk1124 and mk1207 on chromosome 5. The QTL *qol2-5.1*, associated with (*Z*)-3-hexen-1-ol, was also detected in both years; it explained 24.40 and 14.54% of the phenotypic variation for (*Z*)-3-hexen-1-ol in 2019 and 2020, respectively, and was located between mk1168 and mk1207 (Table 2).

C9 aroma compounds contributed to typical cucumber-like qualities. A total of 20 QTLs were identified for C9 aroma compounds (Fig. 4, Table 2), including nonanal, (*E*,*Z*)-2,6-nonadienal, (*E*)-6-nonenal, (*E*)-2-nonenal, (*Z*)-3-nonen-1-ol, (*E*)-6-nonen-1-ol, (*E*,*Z*)-3,6-nonadien-1-ol, and (*E*,*Z*)-2,6-nonadien-1-ol, among which four QTLs, *qol8-2.1*, *qol4-2.1*, *qol6-2.1*, and *qol7-2.1*, associated with (*E*,*Z*)-2,6-nonadien-1-ol, (*Z*)-3-nonen-1-ol, (*E*)-6-nonen-1-ol and (*E*,*Z*)-3,6-nonadien-1-ol, were stably detected in the 2 years. Nine QTLs, *qal3-3.1*, *qal4-2.1*, *qal5-3.1*, *qal6-3.1*, *qal7-3.1*, *qal8-6.1*, *qal9-3.1*, *qal10-3.1*, and *qol4-1.1*, associated with nonanal, (*E*)-4-nonenal, (*E*)-6-nonenal, (*Z*)-2-nonenal, (*Z*, *Z*)-3,6-nonadienal, (*E*)-2-nonenal, (*E*,*Z*)-2,6-nonadienal, (*E*,*E*)-2, 4-nonadienal and (*Z*)-3-nonen-1-ol, were detected in 2019, and seven QTLs, *qal3-3.2*, *qal5-2.1*, *qal5-3.2*, *qal7-3.2*, *qal7-6.1*, *qol3-2.1*, and *qol5-2.1*, associated with nonanal, (*E*)-6-nonenal, (*Z*, *Z*)-3,6-nonadienal, 1-nonanol and 2-nonen-1-ol, were detected in 2020. In the QTLs detected stably in both years, the value of QTL *qol8-2.1* associated with (*E*,*Z*)-2,6-nonadien-1-ol was the highest; it was located between mk190 and mk204 on chromosome 2 and explained 11.76% of the phenotypic variation for (*E*,*Z*)-2,6-nonadien-1-ol in 2019 and 17.53% in 2020. Three QTLs, *qol4-2.1*, *qol6-2.1*, and *qol7-2.1*, associated with (*Z*)-3-nonen-1-ol, (*E*)-6-nonen-1-ol and (*E*,*Z*)-3,6-nonadien-1-ol, were also detected in 2019 and 2020; they were both located between mk191 and mk376 on chromosome 2.

### Nine *CsLOX*-type genes clustered on chromosome 2

The major (*E*,*Z*)-2,6-nonadien-1-ol QTL (*qol8-2.1*) was mapped between mk190 and mk204 on chromosome 2; it had the highest LOD score (10.97) in 2020, and explained 17.53% of the phenotypic variation for (*E*,*Z*)-2,6-nonadien-1-ol (Table 2). (*E*,*Z*)-2,6-Nonadien-1-ol was one of the aroma impact compounds for cucumber fruits, and it was the reduced product of (*E*,*Z*)-2,6-nonadienal formed by the action of ADH. (*E*,*Z*)-2,6-Nonadienal was one of the most important aroma impact compounds with the highest content in cucumber fruits. Therefore, candidate genes in the chromosome 2 region of 19.80–25.53 cM were investigated.

**Figure 5:**
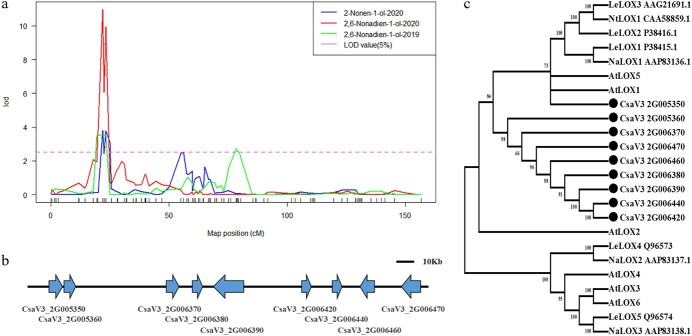
Characterization of a genomic region associated with (*E*,*Z*)-2,6-nonadien-1-ol and (*Z*)-2-nonen-1-ol on chromosome 2. **a** LOD profiles of (*E*,*Z*)-2,6-nonadien-1-ol in the two years and (*Z*)-2-nonen-1-ol in 2020 QTLs detected on the local genetic linkage map of cucumber chromosome 2. **b** Schematic representation of the *LOX* gene cluster. **c** Phylogenetic analysis of nine lipoxygenase genes.

Genes in the lipoxygenase pathway, especially genes for enzymes, were searched in the region from mk190 to mk204 on chromosome 2. The region flanked by these two markers in the cucumber (Chinese Long) V3 reference genome spanned an interval of chromosome 2 from 1 251 767 to 3 966 418 bp and included a total of 381 genes. To search for the candidate gene for (*E*,*Z*)-2,6-nonadien-1-ol, genome-wide *in silico* analysis was performed to detect the functional annotation of genes, especially genes related to the synthesis of aldehydes and alcohols through the lipoxygenase pathway in cucumber. In this QTL region, there were nine lipoxygenase genes located in a physical region of 177 kb, and they were flanked by markers mk190 and mk200 (Fig. 5b).

The nine lipoxygenase genes identified in this region were *CsaV3_2G005350*, *CsaV3_2G005360*, *CsaV3_2G006370*, *CsaV3_2G006380*, *CsaV3_2G006390*, *CsaV3_2G006420*, *CsaV3_2G006440*, *CsaV3_2G006460*, and *CsaV3_2G006470* (Fig. 5b). *CsaV3_2G005350* and *CsaV3_2G005360* were located physically together and within 14 kb, and were upstream of the clustering genes *CsaV3_2G006370*, *CsaV3_2G006380*, and *CsaV3_2G006390*. *CsaV3_2G006420*, *CsaV3_2G006440*, *CsaV3_2G006460*, and *CsaV3_2G006470* were located in a region of 56 kb. To understand the structure and function of the nine lipoxygenase genes, we conducted a phylogenic analysis of the nine cucumber lipoxygenase genes with lipoxygenase genes of other species, including *AtLOX*s from *Arabidopsis*, *LeLOX*s from tomato, and *NtLOX*s from tobacco. This showed that all nine cucumber lipoxygenase genes were in the same clade as *AtLOX1*, *AtLOX5*, and *LeLOX2*, which previously all characterized the *9-LOX* gene (Fig. 5c). The enzymes encoded by the *9-LOX* gene can cleave linolenic or linoleic acid into 9-hydroperoxylinolenic acid.

### Sequence variation of nine lipoxygenase genes

The sequences of the nine lipoxygenase genes were analyzed based on the sequencing dataset of the two parental lines. It was found that there were no significant variations in the sequence of these lipoxygenase genes between the two parental lines except for *CsaV3_2G005360* and *CsaV3_2G006370* (Supplementary Data Table S4). Compared with the CsaV3_2G005360 amino acid sequence in Q16, five amino acid variations (V27L, F114S, S365T, L381V, and R628Q) were found in Q24 (Supplementary Data Fig. S1). Furthermore, an 8-bp insertion was found in the fifth intron in the Q24 DNA genome compared with that of Q16. Twenty-two variable SNPs were detected between the *CsaV3_2G005360* promoters of the parents (Supplementary Data Fig. S2). Nine different *cis*-elements were found between the promoters of the two parental lines, including B3, C2H2, Dof, G2-like, EIL, MYB, TCP, WRKY, and YABBY (Supplementary Data Table S5). *CsaV3_2G005360* encoded a protein with 864 amino acids. There was high sequence similarity between *CsaV3_2G005360* and *CsLOX08* by amino acid sequence alignment of *CsaV3_2G005360* and the predicted lipoxygenase in GenBank. Thus, the *CsaV3_2G005360* gene was named *CsLOX08* in this study. Compared with the coding DNA sequence of *CsaV3_2G006370* in Q16, there were six synonymous SNPs in the coding DNA sequence of Q24. There were 38 variable SNPs and 2 InDels detected between the *CsaV3_2G006370* promoters of the parents. *CsaV3_2G006370* encoded a protein with 860 amino acids. The amino acid sequence alignment of CsaV3_2G006370 and the predicted lipoxygenase in GenBank revealed high sequence similarities between CsaV3_2G006370 and CsLOX06. Thus, the *CsaV3_2G006370* gene was named *CsLOX06*.

### Expression pattern of nine *CsLOX* candidate genes and correlation analysis with aroma compounds

**Figure 6:**
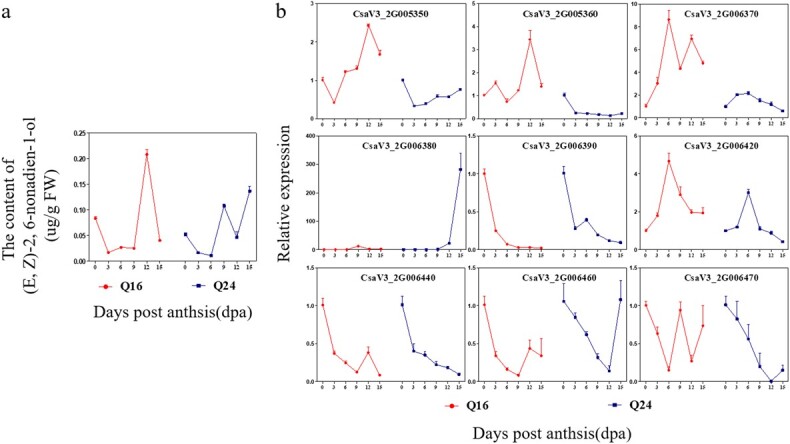
Content of (*E*,*Z*)-2,6-nonadien-1-ol and expression profile of nine *LOX* genes during fruit development in parents. **a** Content of (*E*,*Z*)-2,6-nonadien-1-ol during fruit development in parents. **b** Expression profile of nine *LOX* genes during fruit development in parents. Data are means ± standard error (*n* = 3).

The expression patterns of the nine *CsLOX* genes clustered on chromosome 2 were analyzed at 0, 3,6, 9, 12, and 15 days post-anthesis (dpa) of Q16 and Q24 fruits (Fig. 6b). In Q16, the expression of *CsaV3_2G005350*, *CsLOX08*, *CsLOX06*, *CsaV3_2G005380*, and *CsaV3_2G006420* first increased and then decreased during fruit development, and expression of the other three decreased during fruit development. In Q24, the expression of *CsaV3_2G005370* and *CsaV3_2G006420* first increased and then decreased during fruit development; expression peaked at 6 dpa. The expression of *CsaV3_2G005350* and *CsaV3_2G005380* increased with fruit development, and the expression of others decreased with fruit development in Q24. Aroma compound content was analyzed at 0, 3,6, 9, 12, and 15 dpa of Q16 and Q24 fruits (Fig. 6a). The content of (*E*,*Z*)-2,6-nonadien-1-ol was higher at the late stage of cucumber fruit development and lower at the early stage of cucumber fruit development in the two lines.

**Figure 7:**
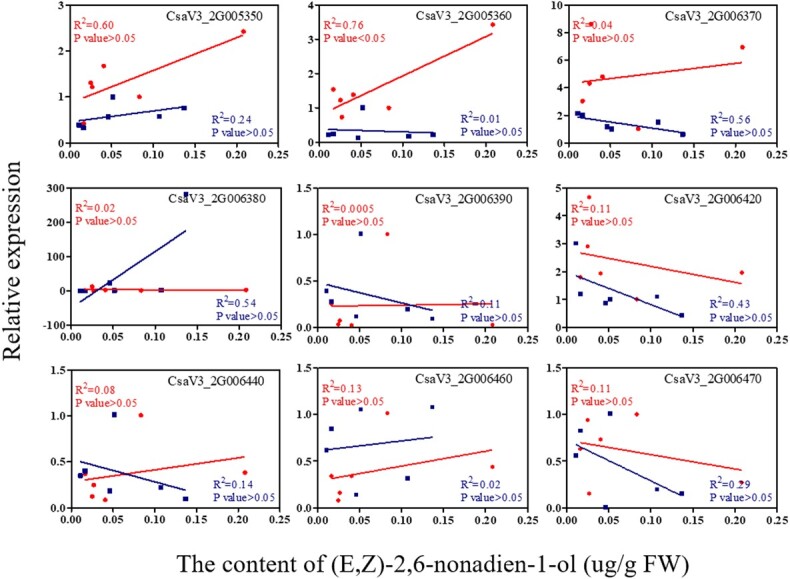
Correlation of (*E*,*Z*)-2,6-nonadien-1-ol content with expression of nine *LOX* genes during fruit development in Q16 (circular symbol) and Q24 (square symbol).

To find which *CsLOX* gene could be associated with the content of (*E*,*Z*)-2,6-nonadien-1-ol, a correlation analysis was performed between the *CsLOX* genes and the content of (*E*,*Z*)-2,6-nonadien-1-ol (Fig. 7a). This showed that the expression of *CsLOX08* was significantly and positively correlated with the content of (*E*,*Z*)-2,6-nonadien-1-ol (*R*^2^ = 0.76, *P* < 0.05) in Q16, but there was no significant correlation between them in Q24. The expression patterns of *CsLOX08* in Q16 and Q24 were different. The expression pattern of *CsLOX08* was the same as the accumulation pattern of (*E*,*Z*)-2,6-nonadien-1-ol in Q16, which was upregulated at stages from 0 to 12 dpa, but expression was low in Q24 at stages from 0 to 12 dpa (Fig. 6b).

### Development of InDel marker and genotyping of RIL population

**Figure 8:**
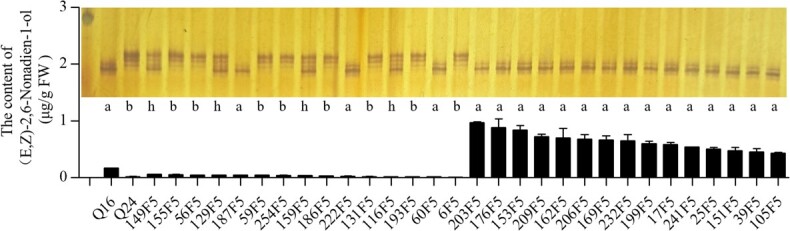
Association between the allelic variation of *CsLOX08* and the content of (*E*,*Z*)-2,6-nonadien-1-ol in extreme individuals from the RIL population. The InDel marker was used to determine the genotypes of *CsLOX08* by polyacrylamide gel electrophoresis of the PCR products. The content of (*E*,*Z*)-2,6-nonadien-1-ol in extreme individuals was analyzed by GC–MS. Data are means ± standard error (*n* = 3).

**Figure 9:**
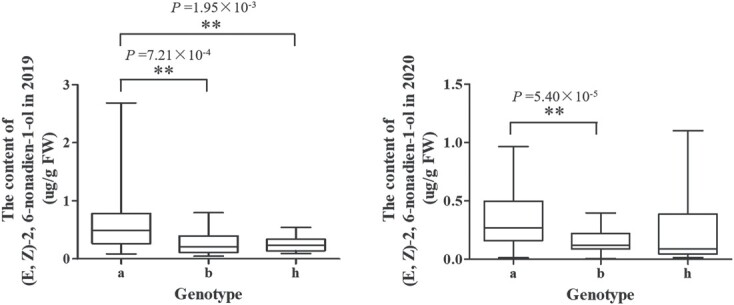
Boxplots showing the association between the genotypes of *CsLOX08* and the content of (*E*,*Z*)-2,6-nonadien-1-ol in the RIL population. The line inside the boxes indicates the median. The top and bottom of the whisker represent the maximum and minimum values, respectively. **P < .01 by Dunnett’s multiple comparison.

According to the 8-bp DNA insertion in the fifth intron of *CsLOX08*, an InDel marker was developed. The RILs were genotyped using the InDel marker, and three genotypes (‘a’, ‘b’ and ‘h’) were present in RILs. Genotypes ‘a’, ‘b’, and ‘h’ stood for dominant homozygous, recessive homozygous, and heterozygous, respectively; ‘a’ represented the loci derived from Q16, ‘b’ represented the loci derived from Q24, and ‘h’ represented loci that were heterozygous. The content of (*E*,*Z*)-2,6-nonadien-1-ol of individuals with the genotype ‘b’ was significantly lower than that of individuals with the genotype ‘a’ in both years (Fig. 9). The genotype results in RIL individuals showed that this InDel marker was closely linked with a high or low content of (*E*,*Z*)-2,6-nonadien-1-ol. Individuals genotyped ‘a’ had a high content of (*E*,*Z*)-2,6-nonadien-1-ol, and individuals genotyped ‘b’ had a low content of (*E*,*Z*)-2,6-nonadien-1-ol (Figs 8 and 9).

### Verification of the InDel marker in some inbred lines

**Figure 10:**
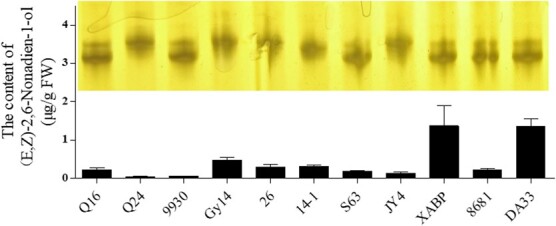
Association between the allelic variation of *CsLOX08* and the content of (*E*,*Z*)-2,6-nonadien-1-ol in nine cucumber inbred lines. The InDel marker was used to determine the genotypes of *CsLOX08* in nine cucumber inbred lines by polyacrylamide gel electrophoresis of the PCR products. The content of (*E*,*Z*)-2,6-nonadien-1-ol was analyzed by GC–MS. Data are means ± standard error (*n* = 3).

Nine cucumber inbred lines were selected to verify the validity of the InDel marker (Fig. 10). Four inbred lines, S63, XABP, 8681, and DA33, which had a higher content of (*E*,*Z*)-2,6-nonadien-1-ol, were identified as genotype ‘a’, and inbred line JY4, which had a lower content of (*E*,*Z*)-2,6-nonadien-1-ol, was identified as genotype ‘b’. However, the genotyping of 9930, Gy14, 26, and 14 was inconsistent with the content of (*E*,*Z*)-2,6-nonadien-1-ol; 9930 was identified as genotype ‘a’ but had a low content of (*E*,*Z*)-2,6-nonadien-1-ol, and Gy14, 26, and 14 were identified as genotype ‘b’ but had a high content of (*E*,*Z*)-2,6-nonadien-1-ol.

## Discussion

### Different kinds and contents of important aroma compounds give fruits distinct flavors

Nineteen characteristic aroma compounds identified by GC–MS were analyzed in parents and RIL populations. All these aroma compounds were known to occur in cucumber fruit, and many were proved to contribute to cucumber fruit flavor [3, 4, 24, 25]. (*E*,*Z*)-2,6-nonadienal and (*E*,*Z*)-2,6-nonadien-1-ol give fruits a typical cucumber-like flavor, which is the most important constituent of the cucumber flavor. Nonanal and nonanol give citrus and rose notes, and (*Z*)-6-nonenal gives melon-like notes [12, 26]. Marked differences in C6 and C9 aroma compound content were found among different genotypes of the RIL population, which was consistent with previous studies in apple and strawberry, where the variation of aroma compounds depends on genotype [27, 28]. C6 and C9 aroma compounds are synthesized by two different branches of the lipoxygenase pathway, in which 13-LOX and 13-HPL catalyze α-linolenic acid and linoleic acid into C6 aroma compounds, and 9-LOX and 9-HPL catalyze α-linolenic acid and linoleic acid into C9 aroma compounds [29]. In both years, significant positive correlations were found among aroma compounds from the same metabolic pathway. For instance, hexanal, 2-hexenal, and 1-hexanol showed significant pairwise correlations in the two years. Significant correlations were also found among C9 aldehydes and alcohols.

### QTLs controlling aroma compounds of cucumber fruit

The content of aroma compounds is a complex quantitative trait controlled by multiple genes and environmental factors. QTL mapping is an efficient tool to screen candidate genes that control quantitative traits. However, few reports have identified the loci that control cucumber aroma compounds using QTL analysis. In our QTL mapping analysis, 28 genomic positions were distributed on six chromosomes (chromosomes 1, 2, 3, 5, 6, and 7) associated with C6 and C9 aroma compounds, and these regions may contain the primary candidate genes that control aroma compound variation. Among them, only 21% of QTLs were reproducible in both years, and the remaining 79% of QTLs were detected in one year. A possible explanation for most QTLs being detected only in one environment is that the aroma compounds of different individuals were affected by the environment in different ways.

QTLs associated with C6 aldehydes and their corresponding alcohols were mainly distributed on chromosome 5, and QTLs associated with C9 aldehydes and their corresponding alcohols were mainly distributed on chromosome 2 and chromosome 3. Several QTL clusters were found on chromosome 2, chromosome 3, and chromosome 5. For example, a QTL cluster (*qol3-2.1*, *qol4-2.1*, *qol6-2.1*, and *qol7-2.1*) associated with C9 alcohols was found on chromosome 2, and another QTL cluster (*qol1-5.1* and *qol2-5.1*) associated with C6 alcohols was found on chromosome 5. The detection of clusters of QTLs could be due to compounds of similar chemical structure being formed from a common biochemical pathway, as had been previously reported in *Arabidopsis* [30], strawberry [31], peach [32], and tomato [33].

### Variation of important aroma compound content may be controlled by lipoxygenase genes

C6 and C9 aroma compounds are synthesized through the lipoxygenase pathway, and lipoxygenases are considered to be the key limiting enzymes controlling the synthesis of C6 and C9 compounds [10, 34]. In our study, a major QTL (*qol8-2.1*) related to (*E*,*Z*)-2,6-nonadien-1-ol was repeatedly detected on chromosome 2, and two consecutive QTL peaks were observed in 2020. So, we speculated that an additional QTL linked to *qol8-2.1* might be present in this QTL locus. Genome scans were performed mainly for the genes involved in the enzymatic action of the lipoxygenase pathway, and a cluster of nine *CsLOX*s was identified in this region. Sequence variation analysis showed that the amino acid sequence of CsLOX08 was polymorphic between two parental lines, and no polymorphism was found in the amino acid sequences of the remaining eight lipoxygenase genes between the two parental lines. Meanwhile, 22 SNPs were detected between the CsLOX08 promoters of the parents. The expression of CsLOX08 was significantly and positively correlated with the content of (*E*,*Z*)-2,6-nonadien-1-ol (*R*^2^ = .76, *P* < .05) in Q16, but there was no significant correlation between them in Q24. We speculated that the variation of CsLOX08 between the two parental lines might be related to the variation of (*E*,*Z*)-2,6-nonadien-1-ol content. Previous studies have shown that *CsLOX08* has an obvious expression signal in flowers, fruits, and roots [35], and the expression of *CsLOX08* in response to the treatments of wounding, ACC, MeJA, ABA, NaCl, and KCl [36]. Five amino acid variations (V27L, F114S, S365T, L381V, and R628Q) were found between the two parental lines. There have been many reports that the amino acid variation of lipoxygenase protein leads to changes in lipoxygenase enzyme activity. For example, the variation of the amino acid (A215V) in *Anabaena* mini-lipoxygenase causes 9-LOX to be converted to 13-LOX [37], and soybean sLOX-1 amino acid change of sF557 was found to increase the activity of the enzyme [38]. So, the amino acid variation of CsLOX08 protein in Q24 may lead to a change in lipoxygenase enzyme activity. The promoter variation of *CsLOX08* in Q16 and Q24 resulted in nine different *cis*-elements between the promoters of the parents, including B3, C2H2, Dof, G2-like, EIL, MYB, TCP, WRKY, and YABBY. The promoter variation in Q16 and Q24 may affect transcription factor binding ability, which leads to different expression levels of *CsLOX08*. Previous studies reported that MYB transcription factors (FaMYB9, FaMYB10, and SlMYB75) and MADS transcription factors (RIN and SlMBP8) directly regulated the lipoxygenase pathway to affect the accumulation of aroma compounds in strawberry and tomato [39–43].

### The molecular marker linked to aroma compound content

The content of aroma compounds was a key factor affecting the flavor of cucumber fruit. Marker-assisted selection (MAS) has been widely used in fruit quality breeding. However, there is no report on molecular markers of aroma compounds in cucumber. In this study, we developed an InDel molecular marker closely linked to (*E*,*Z*)-2,6-nonadien-1-ol. There were two types of InDel markers in the eight cucumber varieties, which indicated that this variation of the *LOX* gene was widespread in natural populations. The genotype pattern of InDel marker and aroma content of several cucumber varieties are not consistent, which may be due to the fact that aroma is a trait controlled by multiple genes.

## Materials and methods

### Plant materials

Two inbred lines, Q16 and Q24, were used in this experiment. Q16 belonged to a Northern China ecotype of cucumber, and Q24 was a landrace cucumber. RILs of the *F*_6_ generation, composed of 148 progeny developed from Q16 × Q24, were used in the autumn of 2019 and spring of 2020 field trials. Nine cucumber inbred lines—9930, Gy14, 26, 14, S63, JY4, XABP, 8681, and DA33—were used in this experiment, and the origin and market class of the nine inbred lines are listed in Supplementary Data Table S6.

### Sampling of plant materials

Plant materials were planted in a plastic tunnel at the Yangling Experimental Demonstration Station (34°31′ N, 107°98′ E) of the College of Horticulture, Northwest A&F University, Yangling, Shaanxi Province, China. Twelve plants of each line were planted.

Q16, Q24, and 129 RILs were planted in the autumn of 2019 and the spring of 2020. Young cucumber leaves were collected and stored at −20°C for DNA extraction. For RILs and parental lines, 12 plants of each line were planted. A total of six to nine well-developed, disease-free fruits from different individuals for each line were sampled at 8–9 a.m. at 12 dpa, and only one fruit was collected per plant. From most lines we collected nine fruits, and the nine fruits were divided into three parts on average, each containing three fruits as a biological repetition. For a few lines we collected six fruits, and the six fruits were also divided into three parts on average, each containing two fruits as a biological repetition. The collected fruits were immediately frozen in liquid nitrogen and then powdered using a grinder (IKA A11 basic, Germany), and stored at −80°C until analysis. Uniform and disease-free fruits from parents at 0, 3, 6, 9, 12, and 15 dpa were collected, and three biological replicates were performed; the fruits were stored at −80°C for GC–MS and gene expression analysis. Uniform and disease-free fruits from nine cucumber inbred lines at 12 dpa were sampled and pooled into three biological replicates for DNA extraction, GC–MS and gene expression analysis.

### Measurement of aroma compound contents

The content of aroma compounds was measured according to Chen *et al*. [11]. The aroma of 5 g frozen fruit powder was extracted by a solid-phase microextraction fiber. After extraction, the contents of the volatile compounds were determined by GC–MS (Thermo Fisher Scientific, Waltham, MA, USA) fitted with an HP-INNWAX column (0.25 mm the inner diameter, 60 m the length, and 0.25 μm the film thickness; Agilent, Shanghai, China).

Authentic standards including hexanal, (*E*)-2-hexenal, 1-hexanol, (*Z*)-3-hexen-1-ol, nonanal, (*E*)-2-nonenal, (*E*,*Z*)-2,6-nonadienal, 1-nonanol, (*Z*)-3-nonen-1-ol, and (*E*)-2-nonen-1-ol were used for qualitative analyses by comparing retention times. The remaining nine aroma compounds were identified according to spectra and retention times which matched entries in the NIST 08 library (NIST and Wiley libraries). The contents of aroma compounds were analyzed by the internal standard method [11]. All samples were analyzed in triplicate.

### SNP identification by genotyping by sequencing and linkage map development

Young leaves collected from Q16, Q24, and 129 RIL individuals were stored at −80°C. Genomic DNA was isolated from leaves using the modified CTAB method [44]. The DNAs of Q16, Q24, and 129 RIL individuals were digested by EcoRI and HaeII restriction enzymes, and then genotyping by sequencing (GBS) libraries were constructed. The GBS library was sequenced using Illumina HiSeq2000 (Novogene, Beijing, China). After filtering the raw sequencing data, high-quality reads were aligned to the cucumber (Chinese Long) reference genome V3 (http://cucurbitgenomics.org/organism/20); the average mapping rate was 98.21% and the average sequence coverage depth was 17.59×. SAMtools was used to detect SNPs from sequencing data. The SNPs were screened by filtering out SNPs that showed distorted segregation (*P* < .001, *χ*^2^ test). Genetic linkage maps were constructed with AsMap [45].

### QTL mapping

QTL mapping was carried out with the R/qtl package (http://rqtl.org/) using the composite interval mapping (CIM) method. The permutation times and significance level were set at 1000 and 0.05, respectively. The QTLs displayed in this study included the chromosome, marker, position, confidence interval, LOD value, and the proportion of aroma phenotypic variance explained by a single QTL (*r*^2^). For each LOD peak, the 1.5-LOD support interval was determined. The QTLs with LOD scores of 3.0 or >3.0 are listed in Table 2. QTLs were named according to chromosome location and trait names, in which ‘*al*’ represents the trait as ‘aldehyde’ and ‘*ol*’ represents ‘alcohol’. The first number refers to the kind of volatile compound, the second number refers to the name of the chromosome, and the third number refers to the number of QTLs. For example, *qol8-2.1* refers to the first QTL for *ol8* [(*E*,*Z*)-2,6-nonadien-1-ol] on chromosome 2, and *qal9-3.1* refers to the first QTL for *al9* [(*E*,*Z*)-2,6-nonadienal] on chromosome 3. QTL positions and 1.5-LOD confidence intervals were drawn using MapChart 2.2 for Windows.

### Screening of candidate genes in *qol8-2.1* region

The candidate genes associated with (*E*,*Z*)-2,6-nonadien-1-ol in the *qol8-2.1* genomic region were screened referring to the cucumber 9930 V3 genome, especially for the genes that participated in the lipoxygenase pathway. To analyze the variation of nine *CsLOXs* between the two parental lines, Q16 and Q24 were re-sequenced on the Illumina sequencing platform by Novogene Co., Ltd (Beijing, China).

The sequences of the *CsaV3_2G006370* and *CsaV3_2G005360* genes were amplified from the cDNA and DNA of Q16 and Q24, respectively, using gene-specific primers (Supplementary Data Table S7), which were cloned into the pMD19-T vector (Takara) and sequenced. A 2000-bp promoter fragment of *CsaV3_2G006370* and *CsaV3_2G005360* was cloned from the genomic DNA of Q16 and Q24, respectively. The promoter fragments were cloned into the pMD19-T vector and sequenced. Sequence alignment was performed using Biowire Jellyfish software (version 1.5, Biowire.com). *CsLOX* promoters were analyzed for transcription factor binding sites (TFBSs) using the PlantTFDB website (http://planttfdb.cbi.pku.edu.cn/). According to the results of sequence alignment, *CsaV3_2G006370* was named *CsLOX06*, and *CsaV3_2G005360* was named *CsLOX08*.

The homologous sequences of *CsLOX*s were searched and downloaded from the NCBI database, and further analysis of the protein sequences was conducted and screened by BLASTP in the cucumber genome database (http://cucurbitgenomics.org/). The redundant sequences and sequences lacking the LOX domain were removed manually. The phylogenetic tree was analyzed by MEGA 5.0 software (http://www.megasoftware.net/) with 1000 bootstrap replications.

### Correlation between candidate gene expression level and alcohol content

Total RNA isolation was performed according to Wan *et al*. [46]. Gene-specific primers for RT–qPCR were designed using Primer 5, and the primer sequence is given in Supplementary Data Table S7. Gene expression analysis of *CsLOX*s was performed using SYBR Green in a BioRad IQ5 PCR thermal cycler (Bio-Rad, USA). The associations between *CsLOX* gene expression and alcohol content were investigated using Graphpad Prism 5.0 (GraphPad Software Inc., La Jolla, CA, USA).

### Polymorphism analysis of candidate genes in parents and InDel marker development

Alignments of amino acid sequences of CsLOXs were performed using ClustalX (version 2.1). A pair of InDel primers was developed based on the 8-bp insertion using Primer Premier 5.0. Polymorphism analysis of InDel markers was carried out by polyacrylamide gel electrophoresis [47].

### Statistical analysis of main aroma compounds

Statistical analysis of compound contents was conducted to estimate the mean, minimum, maximum, and standard deviation in RILs. Homogeneity of variances was tested before ANOVA analyses. Significant differences in aroma compound content between parents were confirmed using Duncan’s multiple range test at a level of *P* < .05. The normality of the distribution of the main aroma compounds in RILs was evaluated by the Shapiro–Wilk test (*P* < .05). The correlation between the main aroma compounds was analyzed using Pearson’s correlation. PCA was employed to analyze the relationship between *CsLOX* expression and aroma compound content during cucumber fruit development. The variables for PCA were normalized by the *Z*-score method. All statistical analyses were carried out using SPSS 22.0 software (Statistical Package for the Social Sciences, Chicago, IL, USA). Violin plots of the distribution of the main aroma compounds in RILs were made using Python programs (3.8 version). Line graphs were generated by GraphPad Prism (version 5; GraphPad Software, USA). The correlation heat map was plotted using MeV software (MultiExperiment Viewer 4.7.4, TIGR, China).

## Supplementary Material

Web_Material_uhac151

## Data Availability

Date are available upon request to the corresponding author.
