# Supplementary material for: Quantitative trait locus mapping of fruit aroma compounds in cucumber (Cucumber sativus L.) based on a recombinant inbred line population
Source: Hortic Res. 2022 Jul 6;9:uhac151. doi: 10.1093/hr/uhac151 (PMC9527598; doi:10.1093/hr/uhac151)
Supplement: Web_Material_uhac151 [file web_material_uhac151.zip › Supplemental Figure.docx]

**Supplemental Material:**


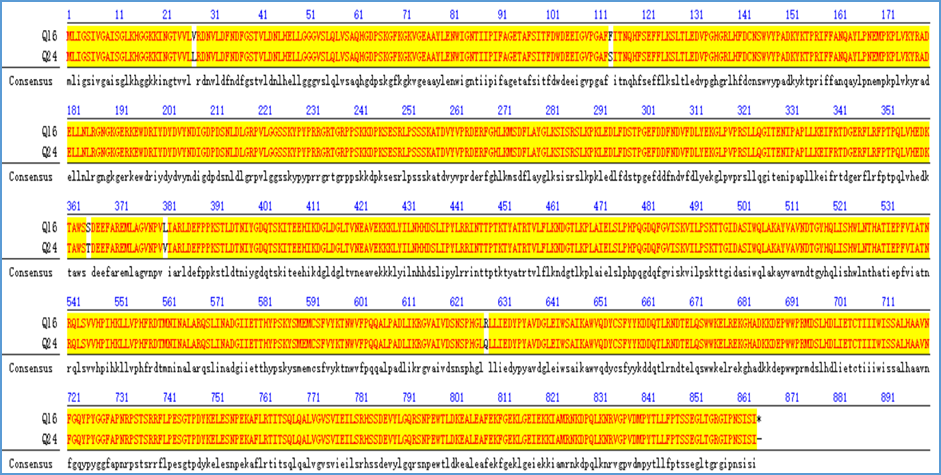


Figure S1: Alignment of the amino acid sequence of *CsLOX08* (*CsaV3_2G005360*) in Q16 and Q24.
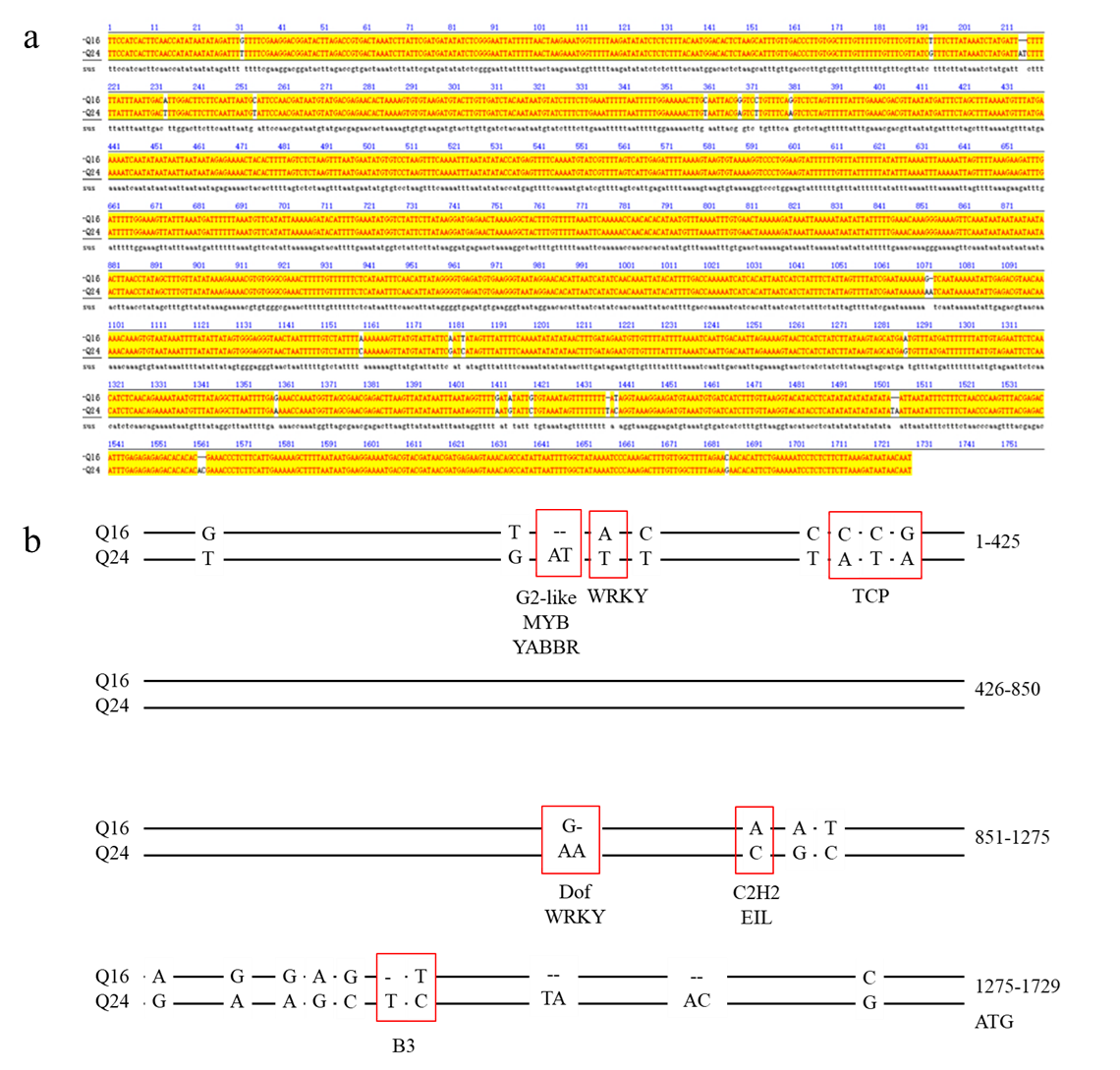
 Figure S2: Promoter analysis of the *CsLOX08* (*CsaV3_2G005360*) from Q16 and Q24.
